# The Combined Inhibitory Effect of the Adenosine A_1_ and Cannabinoid CB_1_ Receptors on cAMP Accumulation in the Hippocampus Is Additive and Independent of A_1_ Receptor Desensitization

**DOI:** 10.1155/2015/872684

**Published:** 2015-01-18

**Authors:** André Serpa, Sara Correia, Joaquim A. Ribeiro, Ana M. Sebastião, José F. Cascalheira

**Affiliations:** ^1^Health Sciences Research Center, University of Beira Interior (CICS-UBI), Avenida Infante D. Henrique, 6200-506 Covilhã, Portugal; ^2^Institute of Pharmacology and Neurosciences, Faculty of Medicine, University of Lisbon, Avenida Professor Egas Moniz, 1649-028 Lisbon, Portugal; ^3^Unit of Neurosciences, Institute of Molecular Medicine, University of Lisbon, Avenida Professor Egas Moniz, 1649-028 Lisbon, Portugal; ^4^Department of Chemistry, University of Beira Interior, Rua Marquês D'Ávila e Bolama, 6201-001 Covilhã, Portugal

## Abstract

Adenosine A_1_ and cannabinoid CB_1_ receptors are highly expressed in hippocampus where they trigger similar transduction pathways. We investigated how the combined acute activation of A_1_ and CB_1_ receptors modulates cAMP accumulation in rat hippocampal slices. The CB_1_ agonist WIN55212-2 (0.3–30 *μ*M) decreased forskolin-stimulated cAMP accumulation with an EC_50_ of 6.6 ± 2.7 *μ*M and an *E*_max⁡_ of 31% ± 2%, whereas for the A_1_ agonist, N^6^-cyclopentyladenosine (CPA, 10–150 nM), an EC_50_ of 35 ± 19 nM, and an *E*_max⁡_ of 29% ± 5 were obtained. The combined inhibitory effect of WIN55212-2 (30 *μ*M) and CPA (100 nM) on cAMP accumulation was 41% ± 6% (*n* = 4), which did not differ (*P* > 0.7) from the sum of the individual effects of each agonist (43% ± 8%) but was different (*P* < 0.05) from the effects of CPA or WIN55212-2 alone. Preincubation with CPA (100 nM) for 95 min caused desensitization of adenosine A_1_ activity, which did not modify the effect of WIN55212-2 (30 *μ*M) on cAMP accumulation. In conclusion, the combined effect of CB_1_ and A_1_ receptors on cAMP formation is additive and CB_1_ receptor activity is not affected by short-term A_1_ receptor desensitization.

## 1. Introduction

Adenosine 3′,5′-cyclic monophosphate (cAMP) is an ubiquitous second messenger which directly activates protein kinase A (PKA) and EPACs (exchange proteins directly activated by cAMP) and opens cyclic nucleotide-gated channels [1, 2]. PKA is the primary downstream effector of cAMP, regulating neurotransmitter release through activation of Ca^2+^ channels or inactivation of K^+^ channels [3, 4]. cAMP is also implicated in memory and synaptic plasticity at the hippocampus through activation of EPACs and through PKA-mediated CREB (cAMP responsive element binding protein) activation [5, 6]. The cAMP signal is transitory and regulated through the opposing actions of adenylyl cyclase and phosphodiesterases [1].

The G_i/o_-protein coupled cannabinoid CB_1_ receptors and adenosine A_1_ receptors are both expressed at high levels in the hippocampus [7, 8], where they inhibit adenylyl cyclase and consequently decrease cAMP production [9, 10]. Furthermore, both receptors colocalize in hippocampal CA3 pyramidal neuron axon terminals, in which they inhibit glutamatergic synaptic transmission to CA1 pyramidal neurons [11–13], are involved in impairment of learning and memory [14, 15], protect against neurotoxic insults, and have antinociceptive action [16–19].

Given the similarity between the transducing pathways operated by adenosine A_1_ and cannabinoid CB_1_ receptors, clarification of the combined activity of these receptors is a particularly interesting issue since both receptors are targets for widely consumed drugs, such as caffeine, an adenosine receptor antagonist, and the psychotropic Δ^9^-tetrahydrocannabinol (THC), a cannabinoid CB_1_ receptor agonist [20]. Interaction between A_1_ and CB_1_ receptors has been reported in* in vivo* studies, where an adenosine A_1_ receptor-mediated enhancement of cannabinoid CB_1_ receptor-induced impairment of short-term spatial memory and motor incoordination were observed [20, 21]. However, the interactions observed* in vivo* might be polysynaptic and dependent on circuitry, not necessarily reflecting receptor interaction at the cellular and molecular levels. Previous studies indicate that when acutely coactivated, adenosine A_1_ and cannabinoid CB_1_ receptors independently inhibit excitatory synaptic transmission in the rat hippocampus and additively stimulate G-protein activation in brain membranes ([12, 22], but see [23]).

Since the putative independence of the acute inhibitory effect of adenosine A_1_ and cannabinoid CB_1_ receptors could be a localized phenomenon, restricted to excitatory synaptic transmission in CA1 area of hippocampus [12], we now further investigated if it also applies to second messenger formation in the whole hippocampus. For that purpose, we studied how the acute coactivation of A_1_ and CB_1_ receptors modulates adenylyl cyclase activity in rat hippocampal slices. Clarification of the combined activity of these receptors on cAMP production would also help to understand how cells integrate signals triggered from both A_1_ and CB_1_ receptors to regulate brain cells activity. On the other hand, since even subchronic activation of A_1_ receptor can induce its desensitization [24] and therefore might cause cross desensitization of the CB_1_ receptor [25], the effect of short-term adenosine A_1_ receptor desensitization on the combined action of adenosine A_1_ and cannabinoid CB_1_ receptors was also investigated.

## 2. Material and Methods

### 2.1. cAMP Accumulation in Hippocampal Slices

The experiments were performed using acute hippocampal slices taken from young adult male Wistar rats (6–8 weeks old). The animals were handled according to European Community guidelines and Portuguese law concerning animal care and were anesthetized with halothane before decapitation. The brain was rapidly removed and transferred to ice-cold Krebs-Henseleit buffer with the following composition (mM): NaCl 118, KCl 4.7, KH_2_PO_4_ 1.2, MgSO_4_ 1.2, CaCl_2_ 1.3, NaHCO_3_ 25, glucose 11.6, gassed with 95% O_2_ and 5% CO_2_ (pH 7.4). The brain was cut longitudinally, the two hippocampi were dissected and cross chopped (350 × 350 *μ*m) with a McIlwain tissue chopper. Sliced hippocampi were then placed in an Erlenmeyer, dispersed, and washed twice with buffer. The cross chopped hippocampal slices were transferred into a conical-bottom polypropylene tube and 50 *μ*L aliquots of gravity-packed slices (1-2 mg protein) were pipetted into flat-bottom propylene tubes (1.65 × 9.5 cm, 20 mL capacity) containing Krebs buffer and preincubated for 30 min at 37°C in a shaking (1 cycle*·*s^−1^) water bath. Since basal intracellular levels of cAMP in hippocampal slices are low and hard to quantify, most experiments were performed in the presence of forskolin and rolipram in order to increase cAMP concentration. Forskolin directly stimulates adenylyl cyclase while rolipram inhibits phosphodiesterase 4, the main enzyme responsible for cAMP degradation in the brain [26]. Incubation with drugs started with addition of rolipram (50 *μ*M final concentration). Forty-five min after rolipram addition, incubation proceeded in the absence or in the presence of forskolin (10 *μ*M) for a further 15–35 min period. Assays performed in the presence of forskolin, and its controls, also contained ethanol (0.02%, v/v), its vehicle. When used, WIN55212-2 (0.3–30 *μ*M), adenosine deaminase (2 U/mL), or DPCPX (50 nM) were present simultaneously with the start of incubation with rolipram, while AM251 (10 *μ*M) was added 30 min before addition of rolipram. CPA (10–150 nM final concentration), when present, was added 30 min after rolipram addition. In one set of experiments WIN55212-2 (30 *μ*M) was present since 5 h and 15 min before rolipram addition and in another set CPA (100 nM final concentration) was added 50 min before rolipram. The final volume after all drug additions was 300 *μ*L. Note that, usually, longer incubation times were used when testing the effect of WIN55212-2 than when testing the CPA effect; this was necessary because WIN55212-2 is very lipophilic and therefore needed longer incubations times to equilibrate with hippocampal slices and produce its inhibitory effect (see [12]). In fact we have found, in a previous electrophysiological study using hippocampal slices [12], that WIN55212-2 started to produce effect on neurotransmission only after 30 min after its application to the hippocampal slice, and it took 60–90 min to produce its maximal effect. When testing the effect of a drug, a parallel control assay was done in which a same volume of vehicle replaced the volume of drug solution added to the tube. Tubes were gassed for 20 s and capped, after slices or drug addition.

Incubations were stopped by adding 100 *μ*L of perchloric acid (HClO_4_, 10% w/v) solution containing EDTA (20 mM). Samples were sonicated for 2 minutes, placed on ice for 30 minutes, neutralized by addition (100 *μ*L) of potassium carbonate (K_2_CO_3_, 0.5 M), and vortexed for 2 minutes, allowing the CO_2_ to escape. The tubes were then placed on ice for an additional 15 minutes period to precipitate potassium perchlorate. The samples were centrifuged (5000 g, 10 min at 4°C) and 200 *μ*L aliquots, per sample, of the supernatants were collected and stored at −80°C for cAMP content analysis. The pellets were digested with NaOH (1 M) for 1.5 h at 37°C, neutralized, and individually assayed in duplicate for protein content by the method of Peterson [27]. The samples were analyzed for cAMP content using an enzyme immunoassay (EIA) kit (Cayman Chemical). cAMP concentration in each sample was expressed as pmol per mg of protein.

### 2.2. Drugs Used

(R)-(+)-[2,3-Dihydro-5-methyl-3-(4-morpholinylmethyl)pyrrolo[1,2,3-de]-1,4-benzoxazin-6-yl]-1-naphthalenylmethanone mesylate (WIN55212-2), 8-cyclopentyl-1,3-dipropylxanthine (DPCPX), N6-cyclopentyladenosine (CPA), N-(piperidin-1-yl)-5-(4-iodophenyl)-1-(2,4-dichlorophenyl)-4-methyl-1H-pyrazole-3-carboxamide (AM251), rolipram, and forskolin were purchased from Tocris (Bristol, UK). Adenosine deaminase was obtained from Sigma-Aldrich. Stock solutions of WIN55212-2 (20 mM), rolipram (20 mM), DPCPX (50 *μ*M), and AM251 (5 mM) were prepared in dimethyl sulfoxide (DMSO). Forskolin (50 mM) and CPA (2 mM) stock solutions were prepared in ethanol and water, respectively. Suitable dilutions of each stock solution with Krebs buffer were made before performing the experiments.

### 2.3. Data Analysis

The values are expressed as mean ± S.E.M. from *n* experiments. The significance of the differences between the mean values obtained in two different conditions, or when comparing means with zero, was evaluated by Student's *t*-test, where the paired Student's *t*-test was used whenever evaluating the significance of differences between two conditions tested in a paired way in the same experiment. When more than two different conditions were simultaneously compared, One-way ANOVA was used followed by the LSD post-hoc test. The maximal effect (*E*_max⁡_) and the concentration of agonist producing half-*E*_max⁡_ (EC_50_) were calculated by fitting the agonist concentration-response curve data to a Michaelis-Menten type equation, through nonlinear regression using the SPSS for Windows program version 16.0 (SPSS Inc., Chicago, Illinois, USA).

## 3. Results

### 3.1. Maximal Effect, Potency, and Specificity of Adenosine A_1_ and Cannabinoid CB_1_ Agonists on Forskolin-Stimulated cAMP Accumulation

In the presence of rolipram (50 *μ*M), the cAMP accumulation was 40 ± 11 pmol/mg protein (*n* = 3), whereas the further addition of 10 *μ*M forskolin increased basal cAMP accumulation by about fivefold (to 202 ± 46 pmol/mg protein, *n* = 3, *P* < 0.05, paired Student's *t*-test).

**Figure 1:**
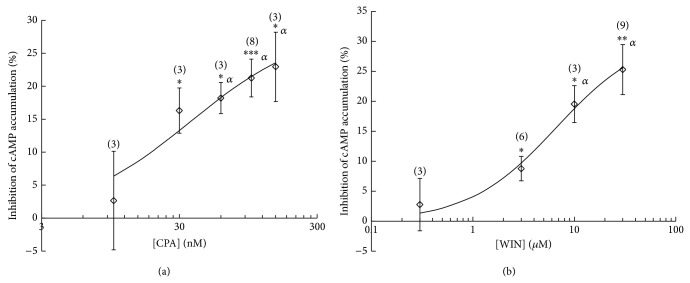
Inhibition of forskolin-stimulated cAMP accumulation by CPA (a) and WIN55212-2 (b) in rat hippocampal slices. (a) Slices were incubated for 30 min in the presence of rolipram (50 *μ*M) and adenosine deaminase (2 U/mL). After this period, incubation continued for a 15 min period in the absence (control) or in the presence of CPA (10–150 nM). Finally incubation proceeded in the presence of forskolin (10 *μ*M) for a further 15 min period. (b) Slices were incubated for 45 min in the presence of rolipram (50 *μ*M) and in the absence (control) or in the presence of WIN55212-2 (0.3–30 *μ*M). After this period, incubation continued for a further 35 min period in the presence of forskolin (10 *μ*M). Data are mean ± SEM of the % inhibition of control cAMP accumulation, corresponding to 3–9 independent experiments run at least in triplicate. The solid lines correspond to the nonlinear regression curves obtained by fitting a Michaelis-Menten type equation to the experimental points. ^*^ *P* < 0.05, ^**^ *P* < 0.01, and ^***^ *P* < 0.001, when compared with zero, Student's *t*-test. ^*α*^Statistically significant (*P* < 0.05) when comparing the cAMP accumulation obtained in the presence of CPA or WIN55212-2 with control cAMP accumulation (One-way ANOVA, followed by LSD test). The number of experiments corresponding to each concentration is indicated in brackets above the bars.

**Figure 2:**
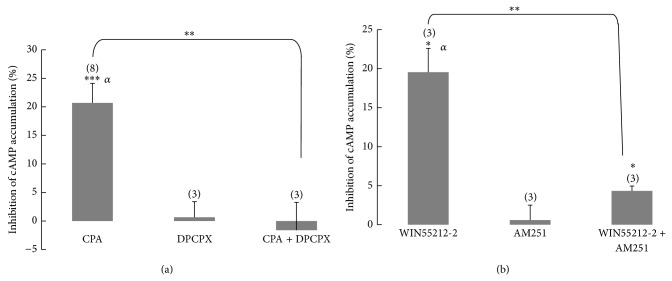
Reversal of the inhibitory effects of CPA (a) and WIN55212-2 (b) on forskolin-stimulated cAMP accumulation, by selective A_1_ receptor and CB_1_ receptor antagonists, respectively. (a) Slices were incubated for 30 min in the presence of rolipram (50 *μ*M), adenosine deaminase (2 U/mL) and in the absence or in the presence of DPCPX (50 nM). After this period, incubation continued for 15 min in the absence or in the presence of CPA (100 nM). Finally incubation proceeded in the presence of forskolin (10 *μ*M) for a further 15 min period. The solid bars represent the % inhibition of control cAMP accumulation produced by (from left to right) CPA, DPCPX, and CPA plus DPCPX. For CPA and for DPCPX the control corresponded to the cAMP accumulation obtained in the absence of both CPA and DPCPX, while for CPA plus DPCPX the control corresponded to the cAMP accumulation obtained in the absence of CPA but in presence of DPCPX. (b) Slices were incubated for 30 min in the absence or in the presence of AM251 (10 *μ*M). After this period, the incubation continued for 45 min in the presence of rolipram (50 *μ*M) and in the absence or in the presence of WIN55212-2 (10 *μ*M). Finally incubation proceeded in the presence of forskolin (10 *μ*M) for a further 35 min period. The solid bars represent the % inhibition of control cAMP accumulation produced by (from left to right) WIN55212-2, AM251, and WIN55212-2 plus AM251. For WIN55212-2 and for AM251 the control corresponded to the cAMP accumulation obtained in the absence of both WIN55212-2 and AM251, while for WIN55212-2 plus AM251 the control corresponded to the cAMP accumulation obtained in the absence of WIN55212-2 but in the presence of AM251. Data are mean ± SEM from 3–8 independent experiments run at least in triplicate. ^*^ *P* < 0.05 and ^***^ *P* < 0.001 when compared with zero (Student's *t*-test). ^**^ *P* < 0.01 when compared with the effect obtained in the absence of antagonist (One-way ANOVA, followed by LSD test). ^*α*^Statistically significant (*P* < 0.05) when comparing the cAMP accumulation obtained in the presence of CPA or WIN55212-2 with control cAMP accumulation (One-way ANOVA, followed by LSD test). The number of experiments performed in each situation is indicated in brackets above the bars.

As shown in Figures 1(a) and 1(b), both the adenosine A_1_ receptor selective agonist CPA (10–150 nM) and the cannabinoid CB_1_ receptor agonist WIN55212.2 (0.3–30 *μ*M) dose-dependently inhibited forskolin-stimulated cAMP accumulation in the hippocampus. Computerized curve fitting to the data shown in Figure 1(a) gave an EC_50_ for CPA of 35 ± 19 nM and a maximal decrease of cAMP accumulation (*E*_max⁡_) of 29% ± 5%, whereas for WIN55212-2 (Figure 1(b)) an EC_50_ of 6.6 ± 2.7 *μ*M and an *E*_max⁡_ of 31% ± 2% were obtained. Application of CPA (100 nM) caused a 21% ± 3% (*n* = 8) inhibition of cAMP accumulation, while when WIN55212-2 (30 *μ*M) was applied, the cAMP accumulation was decreased by 25% ± 4% (*n* = 9). We found these concentrations adequate to test the combined effect of CPA and WIN55212-2 on cAMP accumulation, since with them we obtained a robust effect. The inhibitory effect of CPA (100 nM) on cAMP accumulation was fully blocked by the adenosine A_1_ receptor selective antagonist DPCPX (50 nM; Figure 2(a)), while the inhibitory effect of WIN55212-2 (10 *μ*M) was strongly attenuated by the cannabinoid CB_1_ receptor selective antagonist AM251 (10 *μ*M; Figure 2(b)). Note that in the presence of AM251, WIN55212-2 produced a residual inhibitory effect on cAMP accumulation (4.3 ± 0.6, *n* = 3; Figure 2(b)). The choice of a 10 *μ*M concentration of WIN55212-2 when studying the reversal of its effect by AM251 was determined by the solubility of AM251. Since AM251 is very lipophilic, it is difficult for it to diffuse into the bulk of the slice so that it reaches the right concentration to efficiently inhibit cannabinoid CB_1_ receptors. Thus, the appropriate concentration of AM251, which depends on its low solubility in aqueous buffer, required that the concentration of WIN55212-2 would not surpass 10 *μ*M. Accumulation of cAMP was not affected by either DPCPX or AM251 alone (Figure 2).

### 3.2. Combined Effect of Adenosine A_1_ and Cannabinoid CB_1_ Agonists

**Figure 3:**
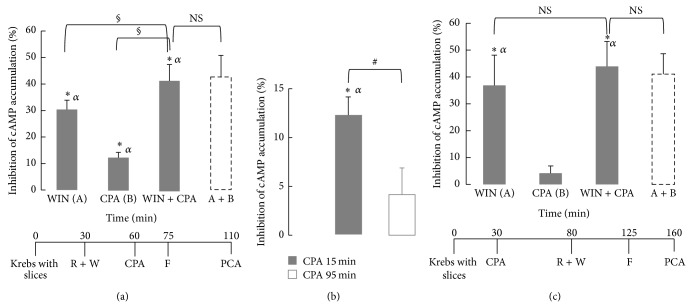
Combined effect of WIN55212-2 and CPA on forskolin-stimulated cAMP accumulation in rat hippocampal slices; influence of the preincubation period with CPA. (a) and (c) Combined effect of WIN55212-2 and CPA. In experiments where CPA was added 95 min before forskolin (c), slices were incubated in the absence (control) or in the presence of CPA (100 nM) for 50 min. After this period incubation continued for 45 min in the presence of rolipram (50 *μ*M) and in the absence (control) or in the presence of WIN55212-2 (30 *μ*M). Then incubation proceeded in the presence of forskolin (10 *μ*M) for a further 35 min period. In experiments where CPA was added 15 min before forskolin (a) CPA (100 nM final concentration) or vehicle (control) was added 30 min after rolipram. In each experiment four parallel assays were performed, corresponding, respectively, to incubation with WIN55212-2, CPA, WIN55212-2 + CPA and incubation in the absence of WIN55212-2 and CPA (control). Solid bars represent the % inhibition of control cAMP accumulation produced by (from left to right) WIN55212-2, CPA, and WIN55212-2 plus CPA; the dashed bar represents the arithmetical sum (calculated for each experiment) of the % inhibition produced by WIN55212-2 and CPA alone. (b) Time-dependent attenuation of the CPA effect. In experiments where CPA was added 95 min before forskolin (open bar), slices were incubated in the absence (control) or in the presence of CPA (100 nM) for 50 min. After this period incubation continued for 45 min in the presence of rolipram (50 *μ*M). Then incubation proceeded in the presence of forskolin (10 *μ*M) for a further 35 min period. In experiments where CPA was added 15 min before forskolin (solid bar) CPA (100 nM final concentration) or vehicle (control) was added 30 min after rolipram. Bars represent the % inhibition produced by CPA of control cAMP accumulation. ^#^Statistically different from CPA added 15 min before forskolin (*P* < 0.05, Student's *t*-test). In the bottom of (a) and (c) are presented the corresponding time lines of addition of drugs. In (b), for CPA 15 min applies the time line presented in (a) and for CPA 95 min applies the timeline presented in (c), but without WIN55212-2. R: rolipram, F: forskolin, W: WIN55212-2, and PCA: perchloric acid. Data are mean ± SEM from 4 independent experiments run at least in triplicate. ^*^Statistically different from zero (*P* < 0.05). ^*α*^Statistically significant (*P* < 0.05) when comparing the cAMP accumulation obtained in the presence of CPA, WIN55212-2, or WIN55212-2 plus CPA, with control cAMP accumulation (One-way ANOVA, followed by LSD test). ^§^Statistically different from the effect of WIN55212-2 (A) or CPA (B) alone (*P* < 0.05; One-way ANOVA, followed by LSD test). NS: the WIN55212-2 plus CPA effect was not statistically different from the sum of the inhibitory effects of WIN55212-2 and CPA alone (A + B, dashed line; *P* > 0.25, when compared within the same experiment, paired Student's *t*-test) in (a) and (c), or from WIN55212-2 alone (A; *P* > 0.07, One-way ANOVA, followed by LSD test) in (c).

When CPA (100 nM) and WIN55212-2 (30 *μ*M) were applied together, respectively, 15 min and 45 min before forskolin, the combined application of WIN55212-2 and CPA produced a higher inhibition of cAMP accumulation (41% ± 6%) than that produced by either WIN55212-2 or CPA alone (Figure 3(a)). Furthermore, the combined effect of CPA and WIN55212-2 did not differ from the sum of the individual effects of each agonist (43% ± 8%; *P* > 0.7, paired Student's *t*-test, Figure 3(a)).

### 3.3. CB_1_ Activity Remains Unaffected by Short-Term Desensitization of Adenosine A_1_ Receptors

As we may observe in Figure 3(b), increasing the preincubation period with CPA, from 15 to 95 min before forskolin addition, caused a significant (*P* < 0.05) attenuation of the CPA effect on forskolin-stimulated cAMP accumulation in the hippocampal slice. In fact, when CPA (100 nM) was applied 95 min before forskolin, no significant effect of CPA was observed (*P* > 0.22, Figures 3(b) and 3(c)) suggesting that short-term desensitization mechanisms were operating on A_1_ receptors. Consequently the possibility that adenosine A_1_ receptor desensitization could cross desensitize cannabinoid CB_1_ receptors and modify the cannabinoid CB_1_-mediated action on cAMP accumulation was investigated. After inducing short-term desensitization of A_1_ receptors by 95 min exposure to CPA, the inhibitory effect of WIN55212-2 (30 *μ*M) on forskolin-stimulated cAMP accumulation was not modified (37% ± 11% inhibition in the absence and 40% ± 13% inhibition in the presence of CPA; *P* > 0.2, paired Student's *t*-test; Figure 3(c)), suggesting absence of cross desensitization of cannabinoid CB_1_ receptors by adenosine A_1_ receptors.

Contrasting with CPA, the WIN55212-2 (30 *μ*M) inhibitory effect on forskolin-stimulated cAMP remained virtually unchanged even when slices were preincubated with WIN55212-2 for up to six hours (31% ± 6% inhibition caused by WIN55212-2 for 45 min preincubation and 30% ± 5% inhibition for 6 h preincubation with WIN55212-2; *P* > 0.05, paired Student's *t*-test). Longer incubation periods were not used to avoid losing slice integrity.

## 4. Discussion

The results obtained in the present work showed for the first time that the inhibitory effect of acute or subchronic coactivation of adenosine A_1_ and cannabinoid CB_1_ receptors on cAMP accumulation is additive in the hippocampus. The results further indicate that the additive inhibitory effects of these receptors are not restricted to excitatory synaptic transmission in the CA1 area [12] but also apply to cAMP formation in the hippocampus. Although a rapid desensitization of the inhibitory action of adenosine A_1_ receptors on cAMP accumulation was observed, this desensitization did not modify the cannabinoid CB_1_ receptor effect on cAMP accumulation.

### 4.1. Potency and Specificity of A_1_ and CB_1_ Agonists as Inhibitors of cAMP Accumulation

The EC_50_ obtained in the present work in the rat hippocampus (36 nM) for the inhibitory effect of the A_1_ receptor selective agonist CPA, when applied 15 min before forskolin, on cAMP accumulation was similar to that obtained in guinea-pig cerebral cortex (22 nM, [28]). The CB_1_ receptor agonist WIN55212-2 potency for inhibition of forskolin-stimulated cAMP accumulation, obtained in the present work (EC_50_ of 6.6 *μ*M), was also similar to that reported for rat* globus pallidus* slices (EC_50_ between 3 and 10 *μ*M, [29]) and slightly higher than that found in mouse cerebellar membranes (EC_50_ of 1.4 *μ*M, [23]). In hippocampal membranes of guinea-pig the effect of WIN55212-2 (7% maximal inhibition, [30]) was very small to calculate the EC_50_. The inhibitory effect of the A_1_ receptor agonist CPA on forskolin-stimulated cAMP accumulation was prevented by the A_1_ receptor selective antagonist DPCPX, indicating that the effect of the agonist was specific for the adenosine A_1_ receptor. The inhibitory effect of WIN55212-2 on forskolin-stimulated cAMP accumulation was strongly attenuated by the CB_1_ receptor selective antagonist AM251, indicating that the WIN55212-2 effect on cAMP accumulation is mainly mediated by cannabinoid CB_1_ receptor. However, even in the presence of AM251, WIN55212-2 produced a small inhibitory effect on cAMP accumulation. This WIN55212-2 residual effect could be due to (i) activation of cannabinoid CB_2_ receptor; (ii) activation of non-CB_1_, non-CB_2_ receptors. Hypothesis (i) seems unlikely since, although WIN55212-2 is not selective for cannabinoid CB_1_ receptor, CB_2_ receptor is mostly found in peripheral tissues. However, hypothesis (ii) cannot be discarded. In fact non-CB_1_, non-CB_2_ activity of WIN55212-2 has been reported in the hippocampus [22, 31], suggesting activation of an unknown receptor.

### 4.2. Combined Actions of A_1_ and CB_1_ Receptors

We quantified cAMP accumulation to determine how adenosine A_1_ and cannabinoid CB_1_ receptors, when coactivated, modulate adenylyl cyclase activity. We found that when both receptors are simultaneously operating, they exert additive inhibition of adenylyl cyclase activity, which implies that the transduction pathways operated by both receptors do not compete or interfere with each other. If both receptors competed for the same limiting pool of adenylyl cyclase, the combined effect of A_1_ and CB_1_ agonists would be less than additive. These findings agree with previous observations obtained concerning hippocampal excitatory synaptic transmission in the rat ([12], but see [32]), in rat hippocampal membranes ([33], but see [20]) and in whole brain membranes of the mouse where coapplication of A_1_ and CB_1_ receptors agonists additively stimulated [^35^S]GTP*γ*S binding [22]. These reports, together with the results obtained in the present study, support an additive effect, in hippocampus, at three different cellular levels when A_1_ and CB_1_ receptors are acutely costimulated: G-proteins, adenylyl cyclase, and excitatory synaptic transmission. In mouse cerebellar membranes, both [^35^S]GTP*γ*S binding and inhibition of forskolin-stimulated cAMP accumulation by combined application of A_1_ and CB_1_ receptors agonists were only partially additive, but still the combined effect was greater than the maximal individual effects [23]. In one study A_1_ receptors attenuate CB_1_ receptor-mediated inhibition of K^+^-induced GABA and glutamate release from rat hippocampal synaptosomes [20], which contrasts with the mutually independent inhibitory action of A_1_ and CB_1_ receptors on hippocampal excitatory synaptic transmission found in brain slices [12], probably because availability of signaling molecules in synaptosomes, shared by both receptors, is lower than in brain slices [34]. In C57BL/6J mice (which have high levels of endogenous adenosine) sustained tonic activation of A_1_ receptors prevented CB_1_-mediated inhibition of excitatory synaptic transmission, but not in the rat [32], suggesting differences between species.

Since adenosine A_1_ and cannabinoid CB_1_ receptors mostly couple to identical G*α*_i/o_ subunits [35] and are both expressed at pyramidal glutamatergic neurons in the hippocampus [36, 37], it is not surprising that receptor interference could occur. In fact, A_1_ receptors have less than additive response when interacting with other G_i/o_-coupled receptors, such as group II metabotropic glutamate receptors [38], *α*_2_-adrenergic receptors [39], and neuropeptide Y receptors [40] in the hippocampus, while, in superior cervical ganglia, the expression of human CB_1_ cannabinoid receptors can sequester G_i/o_ proteins from a common pool and make them unavailable to other G_i/o_-coupled receptors [41]. In rat striatal slices a cannabinoid analogue produced less than additive inhibition of cAMP formation when coapplied with opioid or dopamine D_2_ receptors agonists [42]. On the other hand, additive actions between adenosine A_1_ and *μ*-opioid or GABA_B_ receptor agonists have been described for receptor-mediated G_i/o_ protein activation in hippocampal membranes [33]. Therefore, the additive inhibitory effects of A_1_ and CB_1_ receptors on adenylyl cyclase activity, observed in the present work, suggest that availability not only of G_i/o_ proteins [33], but also of adenylyl cyclase, shared by both receptors, might not be limiting in the rat hippocampus. Another possibility is that compartmentalization of A_1_ and CB_1_ receptors within cells might occur. The scaffold proteins A-kinase anchoring proteins (AKAPs) [43, 44], and the lipid raft caveolae [45], have been identified in the hippocampus, where they improve the spatial precision of cAMP-related activity [46].

Formation of heteromers between adenosine A_2A_ and A_1_ receptors has been reported, which explained the interaction between these two receptors [47]. However, the additive and therefore independent action of A_1_ and CB_1_ receptors at the hippocampus observed in the present work does not suggest formation of heteromers between these two receptors.

### 4.3. Desensitization of A_1_ Receptors

When applied 95 min before forskolin, CPA failed to modify forskolin-stimulated cAMP accumulation. Therefore, 95 min is a sufficient time period to induce subchronic A_1_ receptor homologous desensitization. In fact, rapid (<90 min) homologous desensitization of the A_1_ receptor-mediated inhibition of excitatory neurotransmission, induced by hypoxia, has been reported in the rat hippocampus [48]. In smooth muscle DDT_1_ MF-2 cells, uncoupling of A_1_ receptors from G proteins (measured by a decrease in agonist binding) was observed after 30 min exposure to agonist, an effect involving receptor phosphorylation and arrestin binding [24]. In the same cells, desensitization of the A_1_ receptor-mediated inhibition of forskolin-stimulated adenylyl cyclase activity by preincubation with an adenosine A_1_ receptor agonist takes several hours to occur [49].

Sousa et al. [20] reported unidirectional attenuation by A_1_ receptors of CB_1_ receptor-mediated inhibition of glutamate release from hippocampal synaptosomes, while CB_1_ receptors did not affect the A_1_-mediated effect [20], but in this study CPA was present in the incubation medium before WIN55212-2 for over 30 minutes, which may have been enough to trigger desensitization of A_1_ receptors [24]. To evaluate if this apparent unidirectional action of A_1_ receptors on the CB_1_ receptor-mediated effect could be a consequence of heterologous desensitization by the A_1_ receptors, we studied the influence of the CPA incubation period on the WIN55212-2 inhibitory effect. Addition of CPA either 15 min before forskolin (acute stimulation) or 95 min before forskolin (enough to induce subchronic homologous desensitization) did not modify the inhibitory effect of WIN55212-2 on forskolin-stimulated cAMP accumulation, therefore excluding heterologous desensitization of CB_1_ receptors by acute or subchronic adenosine A_1_ receptor activation, at least at the level of cAMP production. However it does not preclude the hypothesis of heterologous desensitization of the receptor response by longer treatment with receptor agonists [23, 50], where other downstream effectors may be influenced.

Contrasting with adenosine A_1_ receptors, preincubation with WIN55212-2 for up to 6 h did not induce desensitization of the cannabinoid CB_1_ receptor-mediated inhibition of cAMP production. A previous study in cultured hippocampal neurons indicates that an 18 to 24 h exposure to WIN55212-2 was necessary to produce a significant desensitization of the CB_1_ receptor-mediated inhibition of neurotransmission [50].

### 4.4. Conclusion

The results obtained in the present work indicate an additive inhibition of cAMP accumulation by adenosine A_1_ and cannabinoid CB_1_ receptors in the rat hippocampus. Furthermore, the effect of CB_1_ was not affected by subchronic A_1_ receptor desensitization. Therefore, the results suggest that receptor cross talk between adenosine A_1_ and cannabinoid CB_1_ receptors does not play a role on acute inhibitory actions of A_1_ and CB_1_ receptors on cAMP production at the rat hippocampus. Since cAMP plays a central role in regulating multiple brain cell functions, it is likely that other additive actions of adenosine A_1_ and cannabinoid CB_1_ receptors, besides inhibition of glutamatergic neurotransmission, might occur at the hippocampus, where a promising cumulative neuroprotective action against neurotoxic insults may occur, which deserves future investigation.
